# Design and Validation of Additively Manufactured Metallic Cellular Scaffold Structures for Bone Tissue Engineering

**DOI:** 10.3390/ma15093310

**Published:** 2022-05-05

**Authors:** Mohammad O. Al-Barqawi, Benjamin Church, Mythili Thevamaran, Dan J. Thoma, Adeeb Rahman

**Affiliations:** 1 Department of Civil and Environmental Engineering, University of Wisconsin, Milwaukee, WI 53211, USA; adeeb@uwm.edu; 2 Department of Material Science and Engineering, University of Wisconsin, Milwaukee, WI 53211, USA; church@uwm.edu; 3 Department of Material Science and Engineering, University of Wisconsin, Madison, WI 53706, USA; mthevamaran@wisc.edu (M.T.); dthoma@wisc.edu (D.J.T.)

**Keywords:** cellular structures, additive manufacturing, optimization, SS 316L scaffolds

## Abstract

Bone-related defects that cannot heal without significant surgical intervention represent a significant challenge in the orthopedic field. The use of implants for these critical-sized bone defects is being explored to address the limitations of autograft and allograft options. Three-dimensional cellular structures, or bone scaffolds, provide mechanical support and promote bone tissue formation by acting as a template for bone growth. Stress shielding in bones is the reduction in bone density caused by the difference in stiffness between the scaffold and the surrounding bone tissue. This study aimed to reduce the stress shielding and introduce a cellular metal structure to replace defected bone by designing and producing a numerically optimized bone scaffold with an elastic modulus of 15 GPa, which matches the human’s cortical bone modulus. Cubic cell and diagonal cell designs were explored. Strut and cell dimensions were numerically optimized to achieve the desired structural modulus. The resulting scaffold designs were produced from stainless steel using laser powder bed fusion (LPBF). Finite element analysis (FEA) models were validated through compression testing of the printed scaffold designs. The structural configuration of the scaffolds was characterized with scanning electron microscopy (SEM). Cellular struts were found to have minimal internal porosity and rough surfaces. Strut dimensions of the printed scaffolds were found to have variations with the optimized computer-aided design (CAD) models. The experimental results, as expected, were slightly less than FEA results due to structural relative density variations in the scaffolds. Failure of the structures was stretch-dominated for the cubic scaffold and bending-dominated for the diagonal scaffold. The torsional and bending stiffnesses were numerically evaluated and showed higher bending and torsional moduli for the diagonal scaffold. The study successfully contributed to minimizing stress shielding in bone tissue engineering. The study also produced an innovative metal cellular structure that can replace large bone segments anywhere in the human body.

## 1. Introduction

Bone damaged by illness, stress, or disease has regenerative properties allowing it to heal itself as long as the fracture is small. However, bone injuries can reach a size where self-healing will not occur during a patient’s lifetime. These sizes of defect injuries are often the result of extreme trauma, congenital disabilities, or cancer resections and can be difficult to impossible to treat by conventional methods [1]. The current gold standard for treating bone injuries is autograph, which involves using a three-dimensional bone graft substitute taken from a different part of the patient’s body to replace and treat the loss or damage of the bone. However, the use of a bone graft substitute is limited by harvesting difficulties, donor site morbidity, and the clinicians’ ability to contour delicate 3D shapes [2]. Bone tissue engineering (BTE) is an emerging field that will treat critical size bone defects where the conventional current gold standard fails. The goal of BTE is to carry loads, regrow, and repair damaged bone tissues beyond the natural healing capacity of the human body without the use of a bone graft substitute via materials science, biomechanics, molecular development biology, cell biology, and biomedical engineering [3]. In general, three elements are necessary to successfully engineer the biological tissues of a bone. The first required element is the cells that differentiate and form bone tissue (osteogenic cells) along with the proper signaling molecules to induce differentiation. The second element is a biocompatible, porous scaffold conducive to normal cell functions that will allow the cells and tissue to form on and throughout its matrix. Lastly, there must be proper signaling and biomolecules to induce differentiation and sustain the growth of living tissue [4].

A tissue-engineered scaffold is a three-dimensional lightweight, biocompatible structure that provides mechanical support and replicates bone strength and stiffness. In addition, it acts as a template for cell attachment and stimulates tissue formation by mimicking the properties of the extracellular matrix [5]. A biocompatible scaffold for bone tissue engineering requires the building biomaterials to be osteoinductive (the ability of bone-forming cells in the area where the scaffold is implanted to move across a scaffold and regrow bone tissues), porous, and mechanically compatible with native bone. One of the most important BTE scaffold structural characteristics is the pore size, which should be small enough to allow the binding of the cells to the scaffold and large enough to allow cell migration [6]. Until now, no optimum pore size has been achieved. Scaffolds with pore sizes ranging from 300 to 400 microns showed substantial improvement in bone tissue recovery [7]. Pore sizes ranging from 200 to 500 microns have been widely studied [8,9,10], and results showed effective bone ingrowth into the scaffold. Acceptable pore sizes smaller than 200 micros [11,12] and larger than 500 microns [13,14,15] were reported. Pore size as large as 2000 microns has been studied, and acceptable results were presented [16]. A technique for determining the appropriate pore size, strut size, and porosity was presented [17], considering the performance requirements of the scaffold and the limitations of additive manufacturing. The elastic modulus of the BTE scaffold is considered one of the most critical mechanical properties in addition to its compressive strength, which is required to resist load while the bone heals. A stiffness mismatch between the scaffold and the surrounding tissues could cause scaffold failure before healing of the bone if the scaffold is too weak or could cause bone resorption due to the stress shielding effect if the scaffold is too stiff and strong. Stress shielding is defined as the bone weakening and reduction in bone density due to a decrease in the load carried by the bone as a result of stress removal by an implant. Hence, scaffolds should be optimally designed in terms of structural and architectural configurations. The structural scaffold can be designed using any CAD software [17], surface modeling [18,19], or topology optimization [20,21]. The structural configuration of the scaffold can be characterized as a truss, a replicate of polyhedral unit cells in the 3D space (foams), or triply periodic minimal surface.

Many types of unit cells have been investigated in the literature. A library of unit cells has been presented [22,23], consisting of 11 unit cells. Other unit cells such as cubic lattice [24], diamond lattice, truncated cube [25], the truncated octahedron [26], the rhombic dodecahedron [27], and rhombicuboctahedron [27] were explored. At this point, there is no general rule for selecting the best unit cell type to manufacture the BTE scaffold. In addition, four cubic and circular stainless steel lattice geometries were investigated, and the scaffolds with circular geometry were observed to possess the highest mechanical properties [28].

Scaffolds are made from ceramic, polymeric, or metallic materials. Ceramics and polymeric scaffolds are brittle and exhibit weak resistance to high stresses. Metallic scaffolds such as stainless steel (SS), cobalt, and titanium alloys are commonly used as biomaterials due to their excellent corrosion resistance and superior mechanical properties such as compressive strength, fatigue resistance, and fracture toughness. The 316L stainless steel (316LSS) material is widely used as bone implants due to its availability, low cost, and excellent mechanical properties [29]; however, the biggest drawback of the 316LSS scaffolds is that they are not osteoinductive. Surface coating is the most popular strategy to provide osteoinductive characteristics to the 316LSS scaffolds, which has been widely studied. Ceramic material such as hydroxyapatite (HPA) was successfully used as a coating material on SS braids to enhance the osteoinductivity of the SS bone scaffolds [30]. In addition, bilayer coating was successfully applied on 316LSS implants to provide highly proliferated metallic implants for bone regeneration [31]. Silver ion-exchanged zeolite coatings on additively manufactured 316SS scaffolds were successfully prepared to enhance antibacterial activity and biocompatibility [32].

Additive manufacturing (AM)—commonly called 3D printing—is a recent technique in manufacturing 3D objects by building the object layer by layer. After developing the CAD model using any CAD software, the CAD file is converted to an STL (stereolithography) file, which is imported into the 3D printing machine. The machine applies reverse engineering to collect the data from the STL file and then slices the model into layers that can be constructed to build up the model to the final shape. The availability of AM facilitated the construction of 3D complex designs with very low volumes. By utilizing AM technology, BTE scaffolds with precise architectural and structural configurations can be built [33,34,35,36,37]. AM enables the construction of scaffolds with controlled pore size, strut size, cell size, and cell shape.

An optimum scaffold structure, which is designed to exhibit optimum pore size and elastic properties that relate to bone properties, has the promise of loading the bone to similar load levels to those for healthy bone, thus eliminating the stress shielding phenomenon. Such sophisticated scaffold design with large structural complexity can be fabricated using AM. This study aims to design and manufacture an optimized bone tissue scaffold with an elastic modulus that matches the structural modulus of the cortical bone for sufficient bone ingrowth and repair. A similar study has been conducted on open-porous bone scaffold structures made out of Ti-6Al-4V to match the stiffness of cortical bone [38]; however, experimental testing is required to validate the results from the FEA. In addition, plenty of research studies have been conducted to evaluate the mechanical properties of titanium-based alloy scaffolds as well as design scaffolds with properties that are comparable to the human cancellous bone [39,40,41,42]; however, there are minimal optimization studies that target the human cortical bone properties as well as investigate other elastic properties such as bending and shear moduli. In this study, computational modeling was performed and experimentally validated to predict the metal matrix structural properties.

## 2. Material and Methods

### 2.1. BTE Scaffold Material

The material used for this study is stainless steel (SS) 316L since it is one of the most common metallic BTE scaffold materials and is widely used in AM. A comparison of the mechanical properties of 3D printed SS316L between what is reported by the machine manufacturer and what has been published in the literature is represented in Table 1. It is important to note that there is a large variation in the mechanical properties due to variations in process parameters, build orientation, and SS solid material properties [43,44]. Therefore, the mechanical properties of the additively manufactured SS used in the FEA model were experimentally obtained and are shown in Table 1 [45].

### 2.2. Finite Element Analysis Approach

**Figure 1:**
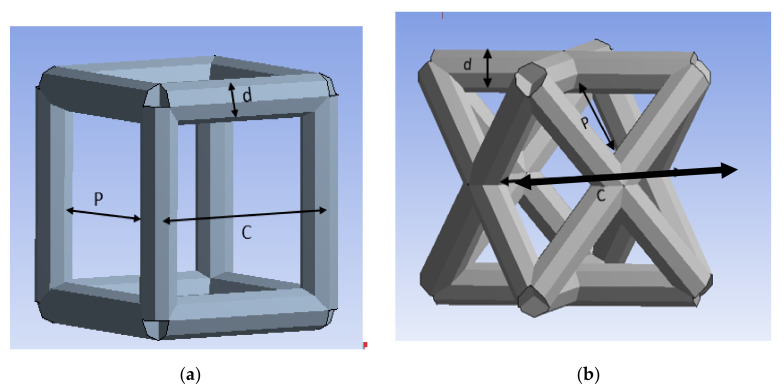
Unit Cells: (**a**) cubic, (**b**) diagonal.

FEA software (ANSYS Workbench) has been used to create the geometry of the BTE scaffold and evaluate its mechanical properties in uniaxial compression. The availability of such FEA tool facilitates the simulation and the prediction of the mechanical behavior of the scaffold without the need to manufacture the scaffold and experimentally test it. Once a target design is achieved, the final product can be manufactured and tested to validate the FE model; hence, significantly saving processing costs. The unit cell types investigated in this study are the cubic and diagonal cell shapes. Each unit call has three geometrical parameters: cell size (*c*), strut size (*d*), and pore size (p), as shown in Figure 1. The pore size (p) was fixed at 800 μm which is sufficient to allow bone ingrowth inside the scaffold, according to studies presented [8,14,47]. The other two geometrical parameters (cell and strut sizes) were parameterized. In this study, the direct optimization tool in ANSYS Workbench is used to obtain optimized geometrical parameters of the unit cell that will provide the BTE scaffold with an elastic modulus of 15 GPa, which is the average structural modulus of the cortical bone.

The relationship between the geometrical parameters *c*, *d*, and p is given through the following equations [38]: 

For cubic unit cell

(1) p=c−d

For diagonal unit cell

(2) $$p=\sqrt{2}\times \left(\frac{c}{2}-d\right)$$

**Figure 2:**
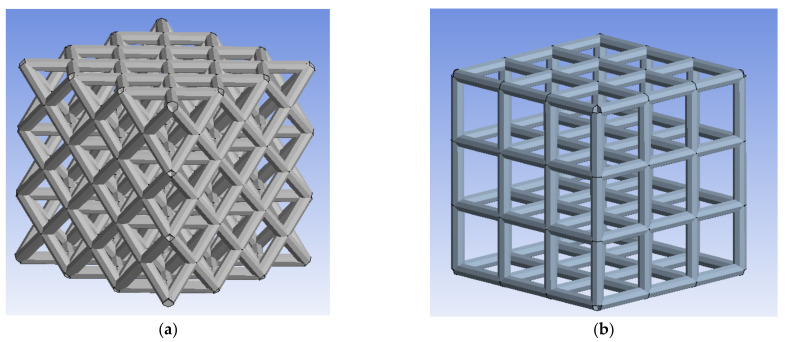
BTE Scaffold: (**a**) diagonal, (**b**) cubic.

The material properties were selected based on tensile test results reported by [45]. To properly characterize the mechanical behavior and deformation of the BTE scaffold under the uniaxial compression load, a beam element was used to mesh the model. The struts in the model will behave like circular structural beams to resist the compression load. Mesh sensitivity analysis was conducted, and it was observed that the number of elements in one strut of the scaffold did not significantly affect the results. Thus, a large mesh size was selected so that each strut contains only one beam element. Figure 2 depicts the mesh for both scaffold designs.

The boundary conditions for the scaffold are inserted in the model by applying zero displacements at the bottom face of the scaffold, which constrains the bottom surface from moving in the direction in which the load is applied. It is also crucial to fully constrain one strut at the bottom of the scaffold by applying a fixed support to prevent the scaffold from undergoing rigid body motion. Applying this configuration of boundary conditions allows the scaffold to move freely in the direction transverse to the loading direction. A linear elastic analysis is run to obtain the Young’s modulus of the cellular scaffold; hence, loading is applied by applying a predefined displacement at the top face of the surface as loading. The displacement value can be arbitrarily selected as long as the analysis is linear elastic.

**Figure 3:**
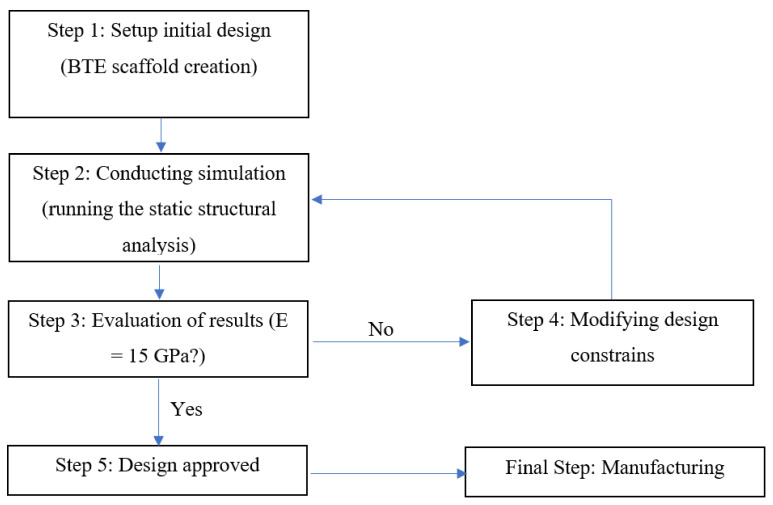
BTE scaffold design optimization process.

A typical engineering optimization process involves the steps shown in Figure 3. The first step is to setup up the initial design, which is the creation of the BTE scaffold with an arbitrary strut size and cell size. According to the AM machine specs used in this research study, the layer thickness of the 3D printed sample can be as small as 300 microns. Hence, the strut size has been constrained within a range of 300–1000 microns. By fixing the pore size to 800 microns, the cell size range for the cubic and diagonal scaffolds can be calculated. Accordingly, the cell size for the cubic scaffold ranges from 1000 to 1800 microns, and for the diagonal scaffold ranges from 1800 to 3200 microns. The cell and strut size are parametrized within their predefined ranges and inserted as design constraints in the optimization process. The second step involves running the simulation using the FE analysis tool after meshing the model, setting up the boundary conditions, applying the loading via predefined vertical displacement in compression, and defining the force reaction at the bottom surface to retrieve the reaction force result. The third step is to evaluate the objective function. The objective function of this optimization is to design a scaffold with an elastic modulus close to the cortical bone’s young’s modulus. The elastic modulus can be calculated using the following equation

(3) $$E_{s}^{*}=\frac{F_{R}\times L_{O}}{A\times \;\delta }$$

where E_s_^*^ is the target elastic modulus of the scaffold, *F_R_* is the force reaction, *L_o_* is the scaffold height, *A* is the enclosed surface area of the scaffold (neglecting pores), and δ is the predefined vertical displacement. If the optimization results are satisfactory, step 5 can be processed, which is design approval. The final step includes manufacturing and testing the final approved design. However, if the results do not agree with the objective function, step 4, which is applying modifications to the design constraints, is required, and another iteration of simulation needs to be conducted. Thus, multiple iterations are required to achieve the final target design. The whole optimization procedure is fully automated in ANSYS Workbench via the direct optimization tool.

### 2.3. Bending and Torsional Stiffnesses

In addition to determining the elastic modulus of the scaffolds under compression, the bending and the torsional stiffnesses of both designs were evaluated. Cantilever beams were created using the results from the optimization and modeled using FEA in bending and torsion. The bending and shear moduli are calculated using the following relationships

(4) $$E_{b}^{*}=\frac{F_{R}\times {L_{O}}^{3}}{3\times I\;\times \delta }.\;$$

(5) $$G^{*}=\frac{T_{R}\times L_{O}}{J\;\times \theta }.\;$$

where *F_R_* is the reaction at the fixed end support, *L_o_* is the beam length, I is the moment of inertia, δ is the deflection at the free end, *T_R_* is the torque reaction at the end support, J is the polar moment of inertia, and θ is the free end rotation.

### 2.4. Additive Manufacturing and Structural Characterization

Following the optimization process and design approval, the finalized design can be manufactured. The cellular scaffold structures were additively manufactured in an EOS M290 printer (Munich, Germany) using Laser Powder Bed Fusion (LPBF) technology. The structures were oriented at an angle of 30 degrees on the 25 cm × 25 cm × 10 cm build platform. The support structure was created in Magics software (version: Materialise Magics 25.01, creator: Fried Vancraen, CEO, Leuven, Belgium) to dissipate the heat from the newly printed layers such that thermally-induced deformation during printing would be minimized. In addition, the support structure was optimized to reduce its amount and build time. 

**Figure 4:**
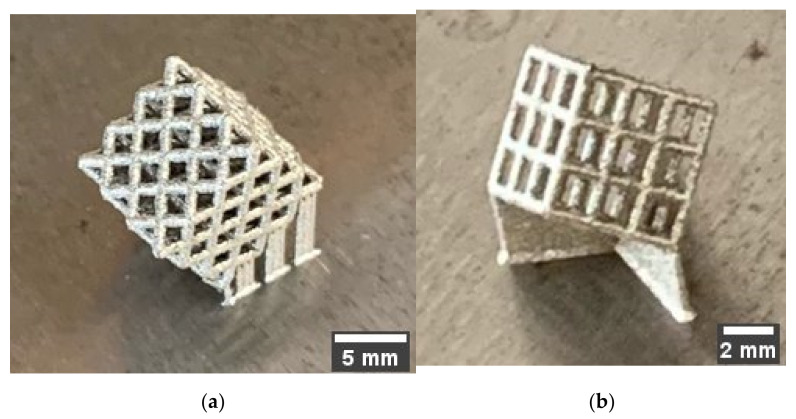
Oriented 3D printed SS scaffolds with support structure: (**a**) diagonal, (**b**) cubic.

**Figure 5:**
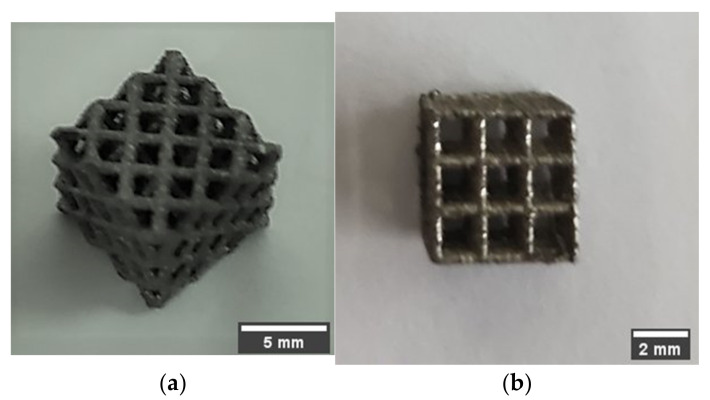
3D printed scaffolds using LPBD after removal of support material and build plate: (**a**) diagonal, (**b**) cubic.

A 400 W Ytterbium fiber laser (EOS, Munich. Germany) with a wavelength of 1060 nm and beam diameter of 100 µm was used for the LPBF processing. Argon gas was used as an inert gas to keep the oxygen as low as 0.1% during printing to prevent oxidation. Gas atomized 316L SS powdered material with a particle size ranging from 15–40 microns was used. The default process parameters used were laser power of 195 W, laser scanning speed of 1083 mm/s, layer thickness of 20 µm, and hatch distance of 0.09 mm. The support structure was sintered in 40 µm layers with a laser power of 100 W and laser speed of 675 mm/s. Electrical Discharge Machining (EDM) was used to remove the scaffold from the substrate. Figure 4 shows 3D printed cubic and diagonal scaffolds on the build plate with a support structure. Figure 5 depicts the 3D printed scaffolds using LPBF after the removal of support material and build plate.

In order to characterize the structural configuration of the 3D printed BTE scaffolds, the samples were analyzed using imaging devices. Standard microscopy, stereo microscopy, and scanning electron microscopy (SEM) were performed. Before imaging, the samples were mounted in clear epoxy with the cube or diagonal lattice facing the polishing surface. Polishing into the face of the scaffold, approximately 1/2 the thickness of the struts parallel to the cube or diagonal scaffold, was performed. Vibratory polishing was performed by using 800-1200-0.5-micron grit. Moreover, the actual struts, unit cells, and pore sizes were measured using SEM and compared with CAD file measurement. The accelerating voltage used in SEM is 10 kV, and images under magnification ranging from 19× to 75× were obtained.

### 2.5. Mechanical Testing and Morphological Characterization

A compression test was conducted on the samples to validate the results obtained from the FE optimization. The compression test was conducted according to ISO 13314 standard, which refers to the mechanical testing of porous and cellular metals. The standard test parameters include sample shape of cylinder or cube, sample dimensions ratio between 1 and 2, and constant strain rate between 0.001/s and 0.01/s (0.06 and 0.6/min). The test was conducted at ambient temperature using a strain rate of 0.005 s^−1^. Height-to-width ratio was 1:1, and specimens’ dimensions were initially measured to the order of 0.1 mm. 

**Figure 6:**
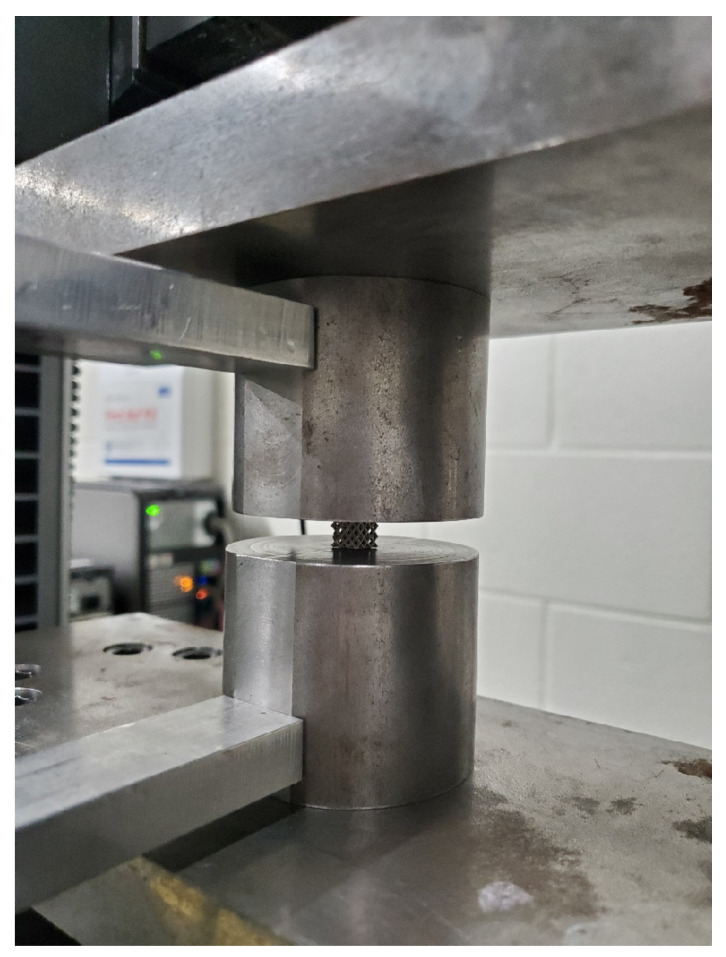
Compressive Testing.

The test device used to conduct the compression test was the 250 kN load cell Instron machine (Instron, Norwood, MA, USA), capable of conducting the test at constant crosshead movement speed. The pressing device consisted of a couple of polished parallel platens used to apply the compressive force on the BTE scaffold. The platens’ geometry was such that the centers of the upper and lower platens could be aligned to the centerline of the machine casing. As for strain measurements, an external LVDT mounted on the platens was used to measure the compressive strain. The external LVDT is very sensitive and is capable of measuring compressive strains with high accuracy. The scaffold was centrally mounted between the platens, in which the centerline of the scaffold coincides with the centerline of the upper and lower platens. The scaffold’s sides that were in contact with the support material were positioned as normal to the platen’s contact surfaces to avoid eccentricity due to surface roughness and irregularity. The force-displacement data for the scaffold were recorded during the testing. The tests continued until the scaffold densification stage in the load-deflection curve was reached. Figure 6 depicts the performed compressive testing.

Scanning electron microscopy (SEM), standard and stereoscopic microscopes were used to morphologically characterize the samples and evaluate the quality of the LPBF technique used to manufacture the scaffolds. Before imaging, the sample was mounted in clear epoxy with the face of the lattice facing the polishing surface. Next, the sample face was polished to approximately half the thickness of the status. Finally, the polishing procedure was accomplished using 800-1200-0.5 micron vibratory. 

The dry weighing and Archimedes techniques were used to determine the structure relative density of the manufactured scaffolds. Dry weighing of the samples was accomplished in normal room temperature and atmospheric conditions. The relative density of the cellular scaffolds was calculated by dividing the measured weight by the theoretical weight of the solid specimen. The theoretical weight was calculated using the theoretical density of the solid SS316L as suggested by the manufacturer, which equals 7.9 g/cm^3^. In the Archimedes technique, the scaffolds were weighed in dry and submerged conditions to the actual volume of the specimens.

## 3. Results and Discussion

### 3.1. Optimization Results and Morphological Properties

The final geometrical parameters generated from the direct optimization tool targeting the structural modulus of the cortical bone are demonstrated in Table 2. 

**Figure 7:**
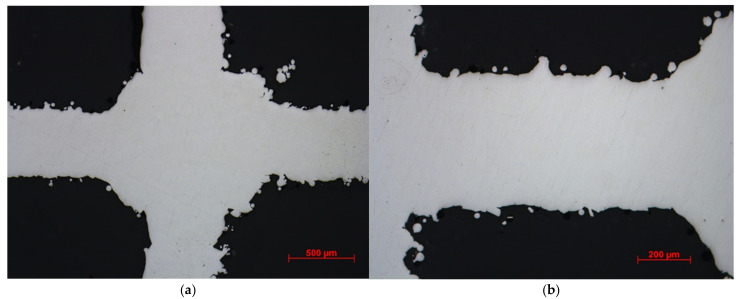
Standard microscopy images at: (**a**) 50× magnification and, (**b**) 100× magnification.

**Figure 8:**
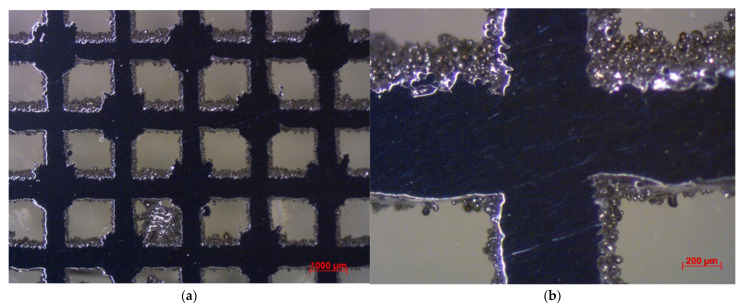
Stereomicroscopy images at: (**a**) 50× magnification and, (**b**) 100× magnification.

**Figure 9:**
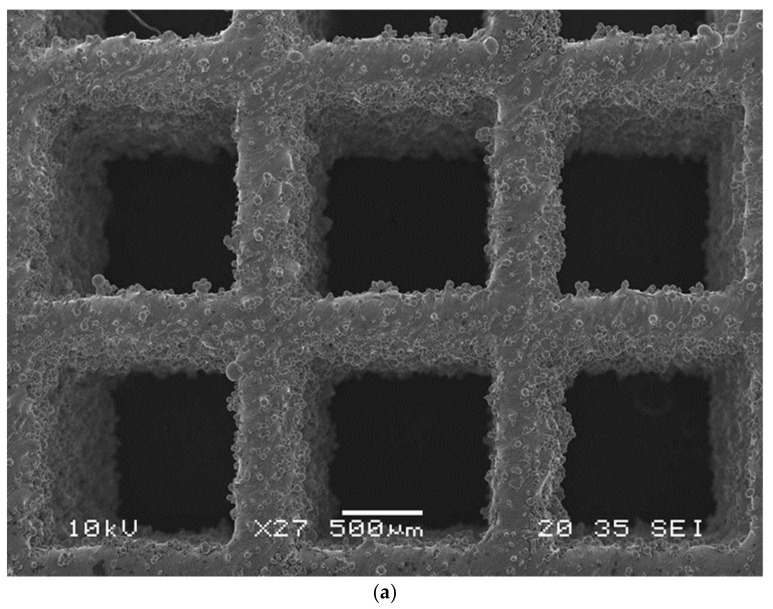

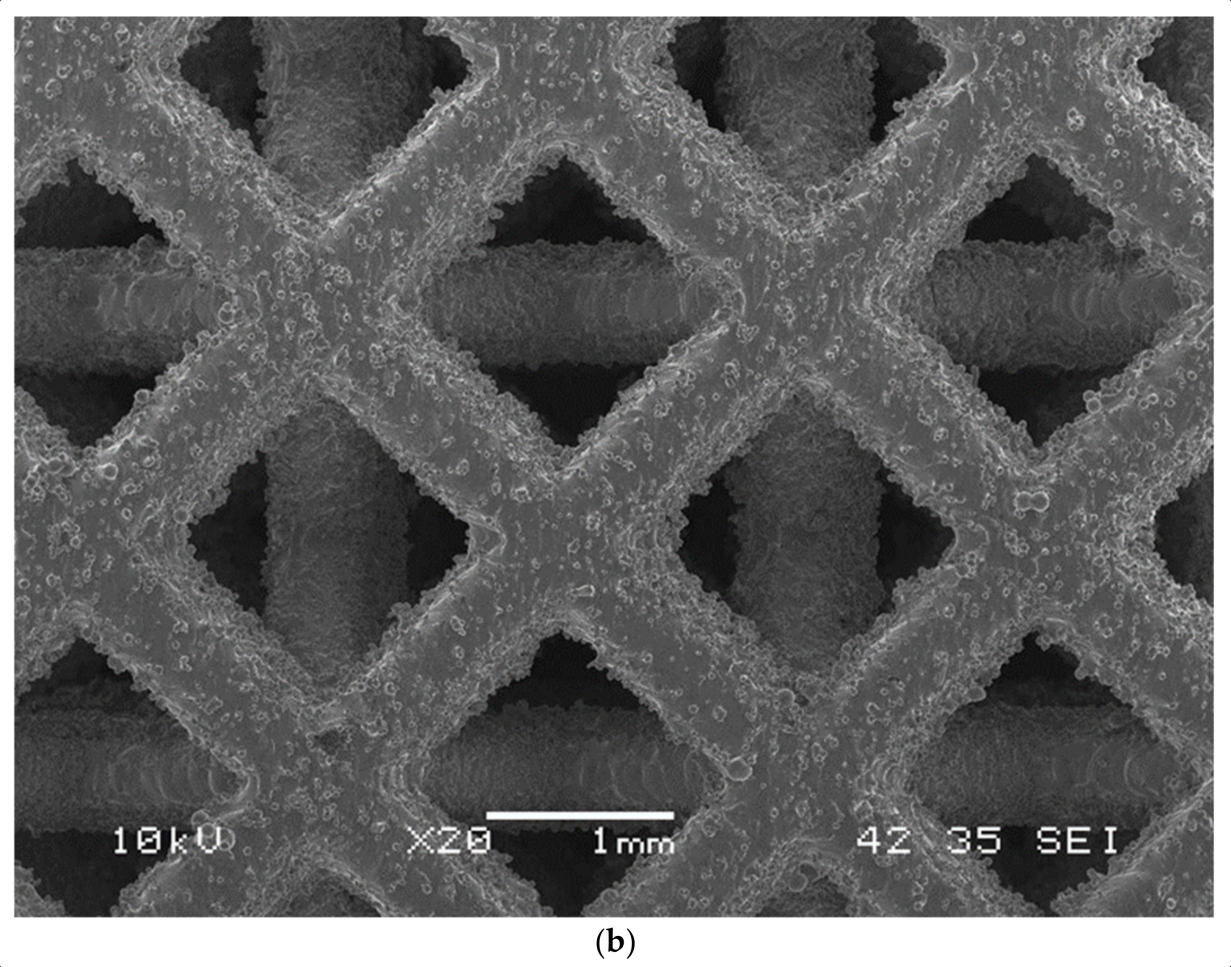
SEM images: (**a**) cubic scaffold, (**b**) diagonal scaffold.

Images from standard and stereo microscopes are shown in Figure 7 and Figure 8. The as-processed stainless steel structures exhibited minimal porosity within the solid struts. However, rough surfaces and edges were noticed, most probably from adhered residual powder on internal features within the LPBF lattice structure. The surface roughness can create potential stress concentration issues on irregular surfaces while testing. In addition, the cross-sectional area of the struts is not constant throughout the scaffold. Instead, the area varies within a specific range, depending on the input parameters used in the additive manufacturing process. Therefore, SEM was used to measure some of the struts’ diameter and basic cell sizes. SEM images of the cubic and diagonal scaffolds are shown in Figure 9. The strut sizes ranged between 597–638 microns for the cubic scaffold and 692–726 microns for the diagonal scaffold. The cell size was measured and found to be around 1439 microns and 2610 microns in both orthogonal directions for the cubic and diagonal scaffolds, respectively, which are very close to the target cell sizes achieved through optimization. However, the average strut sizes for the cubic and diagonal specimens were 3–5% lower than the optimized strut diameter; hence, creating variations in structure relative densities between the CAD file and the manufactured scaffolds; hence, discrepancy in the results is expected. 

Comparisons between the manufactured scaffold structure’s relative density for each unit cell with the optimized scaffold designs are presented in Table 3. The average relative densities for cubic and diagonal scaffolds determined using dry weighing are 32.9% and 35.1%, respectively. The average relative densities for cubic and diagonal scaffolds determined using the Archimedes technique are 33.8% and 36.0%, respectively. The standard deviation is approximately 0.4% for the cubic scaffold and 0.2% for the diagonal scaffolds for both techniques. Comparing the manufactured scaffolds with optimized designs, the percentage errors for the cubic and diagonal designs are approximately 3.2% and 3.4%, respectively.

### 3.2. Validating FEA Results by Experimental Testing

**Figure 10:**
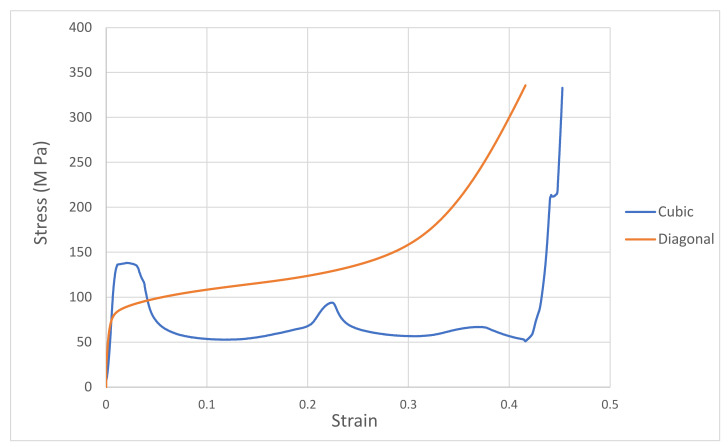
Compressive stress–strain curves for cubic and diagonal scaffolds.

The stress–strain curves for both cubic and diagonal designs are shown in Figure 10. The stress–strain follows a typical stress–strain curve for metallic foams under compression. The curve consists mainly of three regions: (1) linear elastic due to deformation of struts, (2) stress plateau, and (3) densification. The slope of the linear elastic line represents the structural moduli of the cellular scaffold. The elastic moduli generated from the experiment are 14.76 GPa and 14.29 GPa for the cubic and diagonal scaffolds, respectively. The irregularity in strut sizes due to manufacturing explains such a discrepancy between the experimental findings and FEA results in strut sizes as the scaffolds were re-modeled using the actual relative densities, and the elastic moduli values from FEA were in agreement with those experimental values. In addition, FEA assumes idealized material without any defects, thus producing stiffer results. 

Uniform deformation of the vertical struts was observed in the cubic scaffold. The stress fluctuations are related to the failure of the layer by layer mechanism due to the yielding of the struts. Once a layer is fully crushed, the second layer picks up the load, and so on. The post-yield softening regime explains the drop in stress after yielding. Finally, the stress rises steeply at the densification strain when the post-yield ends for the final layer. Such behavior can be related to stretch-dominated structures, which agrees with [48] findings, while for the diagonal scaffold, the whole structure is stressed once loading starts. Initially, the linear elastic behavior is caused by the bending of the struts followed by failure due to the yielding of the struts in each layer. The structure continues to collapse at nearly constant stress (plateau stress) until the cells on the opposite sides are forced into contact, causing the stress to rise steeply at the densification strain. Such behavior is related to bending-dominated structures. The post-yield softening occurring in stretch-dominated scaffolds (cubic) makes them less effective in applications requiring energy absorption. However, they are appropriate for applications that require high load-bearing, which can be explained by the fact that the cubic scaffold has higher compressive strength than the diagonal scaffold. This is because the struts in the cubic scaffold are oriented in the loading direction, making them behave like small columns. The long and flat plateau in bending-dominated scaffolds (diagonal) makes them a better option for energy absorption applications. Diagonal structures produce stable load-carrying capacity with very desirable ductility, which is similar to bone behavior. 

### 3.3. Bending, Shear, and Effect of Porosity

Despite all scaffolds having the same structural modulus, their behavior varies in bending and torsion. The shear and bending stiffnesses of titanium scaffolds were numerically evaluated [38], and their findings agree with the numerical results found in this study. The bending and shear modulus were calculated using the equations shown previously for bending and torsion. The bending modulus for the cubic and diagonal scaffolds are 10.29 GPa and 16.03 GPa, respectively. The shear modulus is 1.39 GPa for the cubic scaffold and 14.52 GPa for the diagonal scaffold. The cubic scaffold has lower bending stiffness than the diagonal scaffold, and much lower torsional bending stiffness, which can be explained by the 45 degrees-oriented struts in the diagonal scaffold carrying the maximum shear stresses in that plane. The cubic design has much less desirable shear resistance. Experimental testing is needed to study the mechanical behavior of the scaffolds under bending and shear.

**Figure 11:**
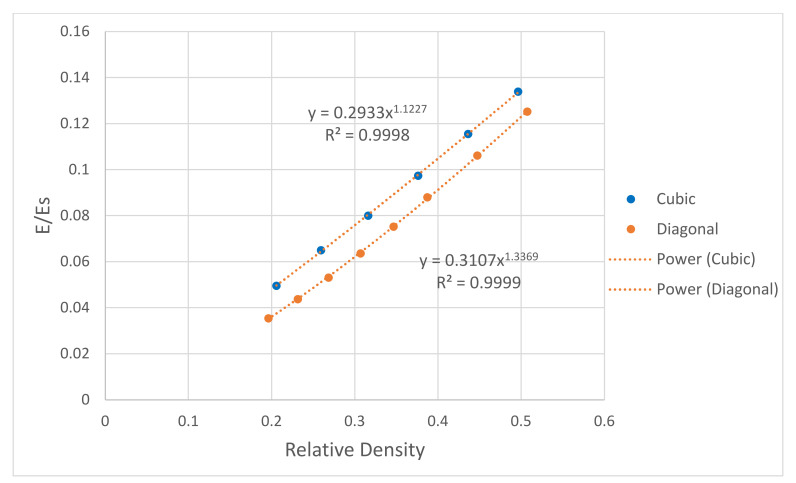
Effect of relative density on scaffold structural modulus.

**Figure 12:**
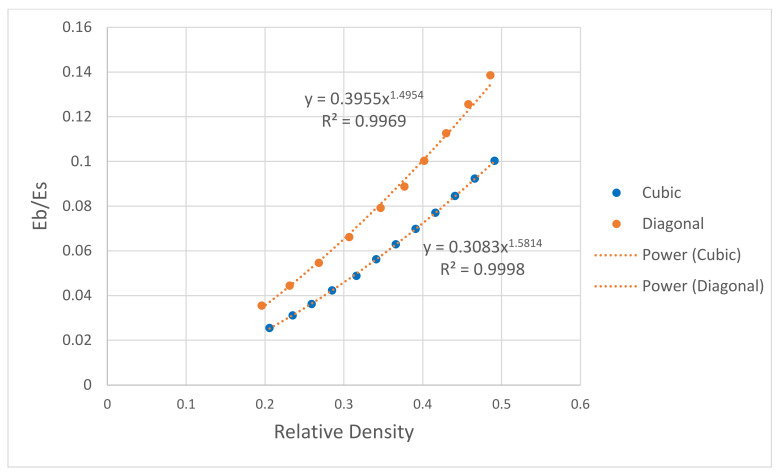
Effect of relative density on scaffold bending modulus.

**Figure 13:**
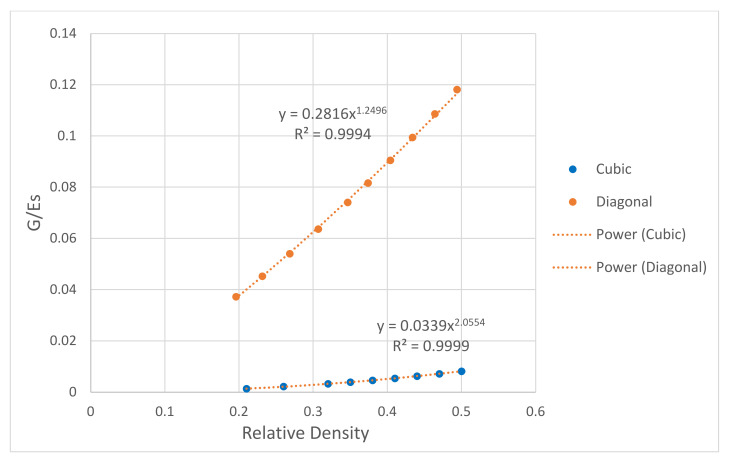
Effect of relative density on scaffold shear modulus.

Moreover, the effect of porosity (1—relative density) on the scaffolds’ compressive, bending, and shear moduli was numerically investigated. The mechanical properties of the scaffolds were normalized to the solid properties of stainless steel presented in Table 1. As expected, the mechanical properties increase with increasing the relative density. All data points were fitted using the power law, and relationships between the mechanical properties of the scaffold and the relative density were generated. For the elastic gradient, the power-law exponent was 1.12 for the cubic and 1.34 for the diagonal scaffold, as shown in Figure 11. Such a finding confirms the stretch-dominated behavior for the cubic scaffold as it is stiffer, and the power exponent is closer to 1, which is lower than the exponent for the diagonal scaffold that exhibits bending-dominated failure. From Figure 12 and Figure 13, the exponents of the power law for the bending stiffness of the cubic and diagonal scaffolds were 1.58 and 1.50, respectively, and the exponents of the power law for the shear stiffness of the cubic and diagonal scaffolds were 1.25 and 2.06, respectively. Thus, the diagonal scaffold exhibits much higher bending and shear stiffnesses than the cubic scaffold. This is also affected by the fact that the diagonal scaffold’s relative density is higher than that for the cubic scaffold. In addition, a slight increase in the bending and shear stiffnesses of the cubic scaffold was observed when increasing the relative density of the cubic scaffold; however, the bending and shear moduli of the diagonal were considerably affected by the scaffold’s structural relative density.

## 4. Conclusions

This research presented the ability to optimize, design, and manufacture bone scaffolds using additive manufacturing with mechanical properties that relate to the cortical bone as part of bone tissue engineering. In addition, the relationship between the morphological and mechanical properties of additively manufactured porous stainless steel scaffolds was investigated. The 316LSS scaffolds exhibit excellent mechanical properties and chemical stability; however, they are not osteoinductive. Hence, coating materials should be applied to provide SS scaffolds with osteoinductive properties, making them suitable for cell growth and cell attachment for bone regeneration.

Optimized geometrical parameters for the cubic and diagonal scaffolds were generated. Experimental testing was accomplished to validate the optimization results. Bending and shear stiffnesses for both scaffolds were evaluated. In addition, the effect of the varying porosity on the elastic properties of the scaffolds was studied. This study has presented a cellular metal structure that can mimic cortical bone strength and stiffness, which has a great potential to be used as bone segment replacement and as a scaffolding structure for bone growth. In addition, the study successfully contributed to minimizing stress shielding in bone tissue engineering using FEA and additive manufacturing of bone scaffolds. Finally, this research highlighted the need to investigate the designed scaffolds’ biomechanical loading behavior and osteointegration properties. Flexural and torsional experiments are needed to capture the full behavior of the optimized scaffolds under bending and shear. 

## Figures and Tables

**Table 1:** Variations in the mechanical properties of 3D printed SS316L between the machine manufacture and values published in the literature [45].

Mechanical Property	From the Manufacturer	From Literature [46]	Values Used [45]
Elastic Modulus (E) GPA	185	188 ± 29	190 ± 45
Ultimate Tensile Strength (UTS) MPa	640 ± 50	592 ± 69	671 ± 33
Yield Strength (YS) MPa	530 ± 60	453 ± 54	560 ± 25
Elongation %	40 ± 15	30 ± 6	24 ± 0.8

**Table 2:** Geometrical parameters of the optimized BTE scaffolds.

Unit Cell Type	Pore Size (Micron)	Cell Size (Micron)	Strut Diameter (Micron)
Cubic	798	1444	646
Diagonal	812	2616	734

**Table 3:** Comparison in structure relative densities between optimized design and manufactured scaffolds.

Unit Cell Design	Optimized Design (%)	Manufactured Scaffold (%)	
		Dry Weighing	Archimedes
Cubic	34.47	32.97	33.77
		33.16	33.98
		32.91	33.85
		33.22	34.12
		32.89	33.91
		32.08	33.04
Diagonal	36.79	35.09	36.16
		35.02	36.10
		35.33	36.29
		34.98	35.83
		35.26	36.21
		34.93	35.68

## Data Availability

Not applicable.
